# Combined Characterization of microRNA and mRNA Profiles Delineates Early Differentiation Pathways of CD133^+^ and CD34^+^ Hematopoietic Stem and Progenitor Cells

**DOI:** 10.1002/stem.627

**Published:** 2011-03-10

**Authors:** Ute Bissels, Stefan Wild, Stefan Tomiuk, Markus Hafner, Hartmut Scheel, Aleksandra Mihailovic, Yeong-Hoon Choi, Thomas Tuschl, Andreas Bosio

**Affiliations:** a Miltenyi Biotec GmbH, Bergisch GladbachGermany; b Howard Hughes Medical Institute, Laboratory of RNA Molecular Biology, Rockefeller UniversityNew York, New York, USA; c Heart Center of the University of Cologne, Department of Cardiothoracic Surgery, Center of Molecular Medicine Cologne, University of CologneCologne, Germany

**Keywords:** CD133, CD34, miRNA copy number, Microarray, Hematopoietic progenitor cells, Hematopoietic stem cell

## Abstract

MicroRNAs (miRNAs) have been shown to play an important role in hematopoiesis. To elucidate the role of miRNAs in the early steps of hematopoiesis, we directly compared donor-matched CD133^+^ cells with the more differentiated CD34^+^CD133^−^ and CD34^−^CD133^−^ cells from bone marrow on the miRNA and mRNA level. Using quantitative whole genome miRNA microarray and sequencing-based profiling, we found that between 109 (CD133^+^) and 216 (CD34^−^CD133^−^) miRNAs were expressed. Quantification revealed that the 25 highest expressed miRNAs accounted for 73% of the total miRNA pool. miR-142-3p was the highest expressed miRNA with up to 2,000 copies per cell in CD34^+^CD133^−^ cells. Eighteen miRNAs were significantly differentially expressed between CD133^+^ and CD34^+^CD133^−^ cells. We analyzed their biological role by examining the coexpression of miRNAs and its bioinformatically predicted mRNA targets and luciferase-based reporter assays. We provide the first evidence for a direct regulation of CD133 by miR-142-3p as well as tropomyosin 1 and frizzled homolog 5 by miR-29a. Overexpression of miRNAs in CD133^+^ cells demonstrated that miR-142-3p has a negative influence on the overall colony-forming ability. In conclusion, the miRNAs expressed differentially between the CD133^+^ and CD34^+^CD133^−^ cells are involved in inhibition of differentiation, prevention of apoptosis, and cytoskeletal remodeling. These results are highly relevant for stem cell-based therapies with CD133^+^ cells and delineate for the first time how the stem cell character of CD133^+^ cells is defined by the expression of specific miRNAs. Stem Cells 2011;29:847–857

## INTRODUCTION

MicroRNAs (miRNAs) are 21–23 nucleotides long noncoding RNAs that are negatively regulating target mRNAs on a post-transcriptional level. Each miRNA has the potential to bind hundreds of mRNAs and one mRNA can be targeted by multiple miRNAs [1], highlighting the importance of miRNAs in complex networks of gene expression regulation. It is estimated that >60% of the mammalian transcriptome is under miRNA control [2]. miRNAs have been shown to play a distinct role in many different cellular, developmental, and physiological processes including hematopoiesis [3].

Detailed analysis of miRNA expression in hematopoiesis showed that miRNAs fine tune essentially each step in hematopoiesis, as summarized in recent reviews [3, 4]. However, most of the performed studies focused on the later steps of hematopoietic lineage differentiation, whereas the early steps of hematopoietic stem cell (HSC) differentiation, for example, the role of miRNAs in self-renewal of long-term and short-term repopulating HSCs are currently mostly unknown. Up to now, only two studies analyzed the expression of miRNAs in primitive human CD34^+^CD38^−^ cells and two recent studies in mouse lineage-negative cKit^+^Sca1^+^ cells [5–8]. miRNA expression profiles are further available for CD34^+^ progenitor cells from bone marrow and mobilized peripheral blood cells [9] as well as CD34^+^ cord blood cells [10].

CD133, also known as prominin-1, was originally found on HSCs and hematopoietic progenitor cells (HPCs) deriving from human fetal liver, bone marrow, peripheral blood, and leukapheresis products from cytokine-mobilized donors [11, 12]. Further studies revealed that long-term culture-initiating cells, the most primitive human hematopoietic cells analyzable in vitro, are highly enriched for CD133^+^ cells [13]. Moreover, CD133^+^ cells in the quiescent phase of the cell cycle have a phenotype consistent with HSCs and have high repopulating activity [14]. As CD34^+^ cells can be generated in vitro from CD133^+^CD34^−^cells [15], CD133^+^ cells appear to be ancestral to CD34^+^cells. Gene expression profiles and repopulation assays provided further evidence for a more primitive phenotype of CD133^+^ cells compared with CD34^+^ cells [16, 17].

In stem cell-based therapies, CD133^+^ cells are used for treatment of leukemia [18, 19], neurodegenerative diseases [20], liver regeneration [21], and myocardial infarction [22, 23]. Currently, the intramyocardial transplantation of CD133^+^ cells is evaluated in clinical trials such as the INSTEM [24] and PERFECT [25] trial.

To elucidate the molecular differences that account for the more primitive character and therapeutic potential of CD133^+^ cells as compared with CD34^+^ cells, we aimed at a comprehensive miRNA expression analysis of both subpopulations.

Recently, we described a microarray-based approach for global and absolute quantification of miRNAs [26]. Here, we present the first relative and absolute miRNA profile of CD133^+^ bone marrow cells and the first comparison of donor-matched CD133^+^ and CD34^+^CD133^−^ cells on miRNA level. The biological role of the miRNAs differentially expressed between CD133^+^ and CD34^+^CD133^−^ cells was further analyzed by examining the coexpression of bioinformatically predicted miRNA-mRNA pairs and the influence of miRNA overexpression in CD133^+^ cells.

## MATERIALS AND METHODS

### Cells and Cell Sorting

HSCs were isolated from human bone marrow following written consent from the local Ethics Committee. The mean donor age was 79 years. CD133^+^ bone marrow cells were enriched with the CliniMACS CD133 Reagent (clone AC133, Miltenyi Biotec, Bergisch Gladbach, Germany, http://www.miltenyibiotec.com) using the CliniMACS Cell Separation System (Miltenyi Biotec). CD34^+^/CD133^−^ cells were isolated by enrichment of CD34^+^ cells with the CliniMACS CD34 Reagent (clone QBEND/10, Miltenyi Biotec) using the negative fraction of the first separation. The gating of the separated cells was performed according to ISHAGE. CD133^+^ cells from mobilized peripheral blood were obtained from AllCells LLC.

### RNA Sources

Total RNA was isolated from the different hematopoietic subpopulations using miRNeasy Mini (Qiagen, Hilden, Germany, http://www.qiagen.com). RNA quality was confirmed using an RNA 6000 Pico total RNA kit (Agilent Technologies, Böblingen, Germany, http://www.agilent.com). RNA integrity numbers were between 8.2 and 9.4.

The universal reference (UR; miRXplore Universal Reference, Miltenyi), consisting of a pool of 954 miRNAs, with each individual oligoribonucleotide having a final concentration of 5 fmol/μl, has been described in detail [26].

### RNA Processing

For miRNA profiling, RNA samples were complemented with miRControl 3 and labeled using the miRCury Power Labeling Kit (Exiqon, Vedbaek, Denmark, http://www.exiqon.com). Hybridization of miRXplore microarrays was performed as described elsewhere [26] using an automated hybridization instrument (a-Hyb Hybridization Station, Miltenyi Biotec).

For mRNA profiling, mRNA was amplified using the μMACS SuperAmp Kit (Miltenyi Biotec) and hybridized on Agilent whole genome arrays.

### Microarray Design and Analysis

The miRNA microarray design of the miRXplore microarrays (Miltenyi Biotec) has been described in detail previously [26]. The exact configuration of each microarray is deposited at the National Center for Biotechnology Information Gene Expression Omnibus (GEO; accession number GSE22460). Analysis of the microarrays was done as described before [26, 27].

For Agilent whole genome arrays, scanned images were analyzed using the Agilent Feature Extraction software (Version 9.1) by which the local background was subtracted. A two-tailed *t* test was used to determine the signal versus background significance. The significance values were used for filtering of the data set, considering only those signals with *p* < .01 on at least three of eight arrays (CD133 and CD34 samples) or two of four arrays (Neg samples). After that, the signal intensity data was normalized to the array median and multiplied by the median calculated from all values.

For downstream statistical analysis of miRNA and mRNA array data, ratios were log 2 transformed and data imported into the MeV program, which is part of the TM4 package [28]. Two-dimensional hierarchical clustering was done using Euclidean distance. The statistical analysis of microarrays was performed using significance analysis of microarrays (SAM) with at least 100 permutations per analysis, *t* test with *p* < .05 and adjusted Bonferroni correction or *t* test with *p* < .01.

### Quantitative Real-Time Polymerase Chain Reaction and miRNA Cloning

The miScript PCR System (Qiagen) was used to validate miRNAs detected as differentially expressed on microarrays. The real-time polymerase chain reaction (RT-PCR) reaction and the real-time PCR were carried out according to the manufacturer's protocol. Data were normalized using RNU6B (MS00014000) or RN5S1(MS00007574). PCR reactions were performed in triplicates and carried out in an ABI Prism 7000 SDS Real Time PCR machine (Applied Biosystems, Foster City, CA, http://www.appliedbiosystems.com) and analyzed using ABI Prism 7000 System SDS software (version 1.1).

Small RNA cDNA library preparation procedure was performed with 2 μg total RNA as input following the basic protocol described in Hafner et al. [29].

### miRNA Target Prediction

Prediction of miRNA targets was performed using TargetScan [30], miRDB [31], PicTar [32], ElMMo [33], miRanda [34], and PITA [35]. For further analysis, targets either predicted by at least three of six target prediction tools or targets predicted exclusively by TargetScan were used.

Combined analysis of miRNA and mRNA profiles was carried out by investigating the coexpression of the bioinformatically predicted miRNA-mRNA pairs. The predicted targets were selected based on [1] their expression level and/or [2] based on inversed expression, for example, miRNA upregulated and the predicted mRNA downregulated.

The expressed (detected) signals were filtered as follows: probes with a *p* below .01 on 75% of the arrays were considered, excluding probes being present in the lowest quartile of the array or if it was not detected on more than 75% of the arrays.

The predicted and coexpressed mRNAs were tested for a significant enrichment of annotations using the proprietary TreeRanker software (Miltenyi Biotec). As annotation sources, databases containing information on gene ontology (GO) categories, protein sequence motifs, interaction data, complex membership, and involvement in biological pathways were used. As background for the analysis, the probe set of the filtered Agilent Whole Human Genome Oligo Microarray was chosen. Enrichment *p* values were computed by Fisher's exact test with subsequent correction for multiple testing using Benjamini Hochberg FDR. The Enrichment Factor is a parameter that shows the extent to which genes in an annotation group are overrepresented, for example, if A of B analyzed genes are annotated as “category X” and overall C of D genes on the whole microarray are annotated as “category X”, the enrichment factor is (A/B)/(C/D). The *p* value threshold was .05 and the factor of enrichment was required to be at least 3.

### Luciferase Assays

60mer DNA oligonucleotides (Metabion, Martinsried, Germany, http://www.metabion.com) consisting of the test sequence (FZD5: AAACTACATATGGCCAAGGTCACTTCCG TTTACCTTCAT GGTGCTGTTGCCCCCTCCCC; tropomyosin 1 (TPM1): AAAC TACATATGTGTTGGAAACACAATCAGGTGTGGATTGGTGC TACTTTGAACAAAAC; CD133: AAACTAGCGGCCGCACTT TTTTACACTGAGT TTCTATTTAGACACTACAACATATGGG GTGC) flanked by PmeI, XhoI (overhangs), and NdeI or NotI (internal) restriction sites were cloned downstream of the firefly luciferase gene into the pmirGLO dual-luciferase miRNA Target Expression Vector (Promega, Madison, WI, http://www.promega.com) using standard procedures.

HEK293T cells were plated into 96-well plates and cotransfected with the described luciferase reporter construct and the appropriate miRNA precursor (Applied Biosystems) using Attractene (Qiagen). Lumincescence was quantitated 48 hours after transfection by using the Dual Glo Luciferase Assay System (Promega) on a GENios Reader (Tecan, Männedorf, Switzerland, http://www.tecan.com).

### Cell Division Tracking and Colony-Forming Unit Assays

CD133+ cells were cultivated in StemSpan serum-free medium (StemCell Technologies, Vancouver, Canada, http://www.stemcell.com) supplemented with 10 μg/ml heparin (Ratiopharm, Ulm, Germany, www1.ratiopharm.com), 10 ng/ml mouse SCF (R&D Systems), 20 ng/ml mouse TPO (R&D Systems, Minneapolis, MN, http://www.rndsystems.com), and 10 ng/ml human FGF-1 (Invitrogen, Frederick, MD, http://www.invitrogen.com). Cells were cultured at 37°C in 5% CO_2_ in U-bottom 96-well plates with 150 μl of the indicated medium. The number of cell divisions were analyzed using carboxyfluorescein diacetate *N*-succinimidyl ester (CFSE; Sigma-Aldrich, Steinheim, Germany, http://www.sigmaaldrich.com) labeling as described by Walenda et al. [36]. One day after CFSE labeling the CD133+ cells were transfected with appropriate miRNA precursor (Applied Biosystems). Transfection was performed by using HiPerfect (Qiagen) according to the manufacturer's protocol.

For colony-forming unit (CFU) assays, transfected cells added to methylcellulose media (HSC-CFU complete with Epo; Miltenyi) and plated in 6-well plates at a final concentration of 150 CD34^+^cells per milliliter. After incubation for 14 days at 37°C and 5% CO_2_ colonies were classified by their color and morphology using an inverted microscope.

## RESULTS

### Relative and Absolute miRNA Expression in CD133^+^ Cells

**Figure 1:**
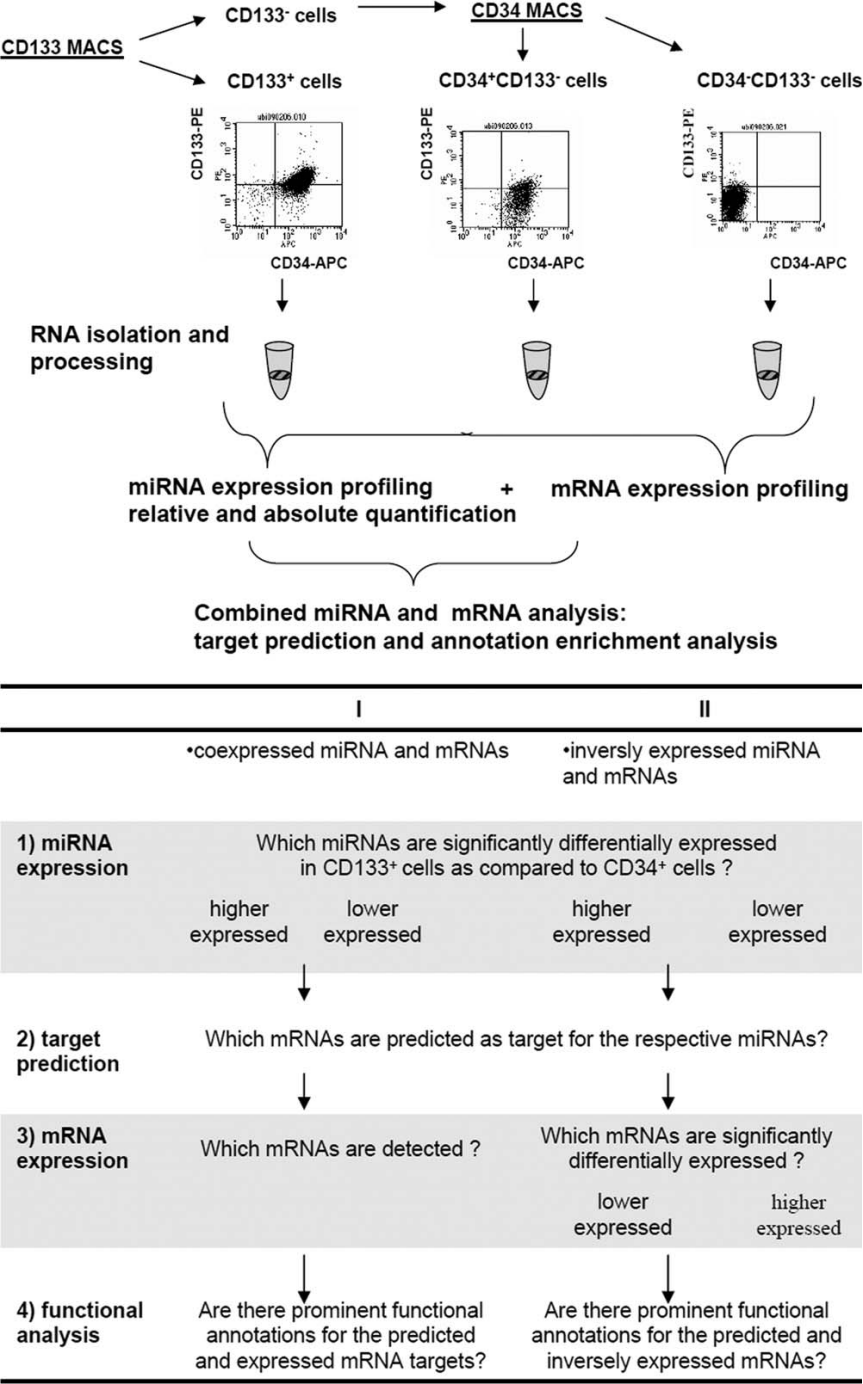
Experimental procedure for miRNA expression analysis in hematopoietic stem cells and hematopoietic progenitor cells. (**A**): After separation of CD133^+^, CD34^+^CD133^−^, and CD34^−^CD133^−^ cells, the RNA was isolated and further processed for miRNA or mRNA profiling. Hybridization was carried out using miRXplore microarrays for miRNA analysis and Agilent Whole Human Genome Oligo Microarrays for mRNA analysis. Subsequently, the miRNA and mRNA profiles were used to elucidate the biological function of the significantly differentially expressed miRNAs. (**B**): Detailed workflow for the combined miRNA and mRNA analysis. Abbreviations: miRNA, microRNAS.

CD133^+^ cells were magnetically isolated from bone marrow by MACS technology. Subsequently, CD34^+^CD133^−^ cells were isolated using the negative fraction of the first separation (Fig. 1A). Besides the CD34^+^CD133^−^ cell population, CD34^−^CD133^−^ cells (the negative fraction of the second separation) were also analyzed and compared with CD133^+^ cells to get a general impression of miRNA expression in bone marrow cells. As the main cell type present in the CD34^−^CD133^−^ fraction are erythrocytes, an additional analysis of CD34^−^CD133^−^ cells after red blood cell (RBC) lysis was performed (Neg −R). Here, the main cell population is neutrophilic granulocytes [37].

RNA samples from the bone marrow subpopulations, that is, CD133^+^, CD34^+^CD133^−^, CD133^−^CD34^−^ (Neg +R), and CD133^−^CD34^−^ (Neg −R), of five different donors and additionally two samples of CD133^+^ cells from mobilized leukapheresis (Supporting Information Table 1) were labeled with Cy5 and hybridized versus the Cy3-labeled miRXplore Universal Reference. The UR is an equimolar pool of 954 synthetic miRNAs of known concentration that allows direct comparison of miRNA expression across multiple experiments and absolute quantification [26].

The overall number of detected miRNAs (in at least four of five donors) was 109 in CD133^+^ cells, compared with 126 miRNAs in CD34^+^CD133^−^ cells (Supporting Information Fig. 1), 151 miRNAs in CD133^−^CD34^−^ cells without RBCs (Neg –R), and 216 miRNAs in CD133^−^CD34^−^ cells with RBCs (Neg +R). The diversity of miRNAs was highest in the CD133^−^CD34^−^ cell population including RBCs and lowest in CD133^+^ and CD34^+^CD133^−^ cells.

**Figure 2:**
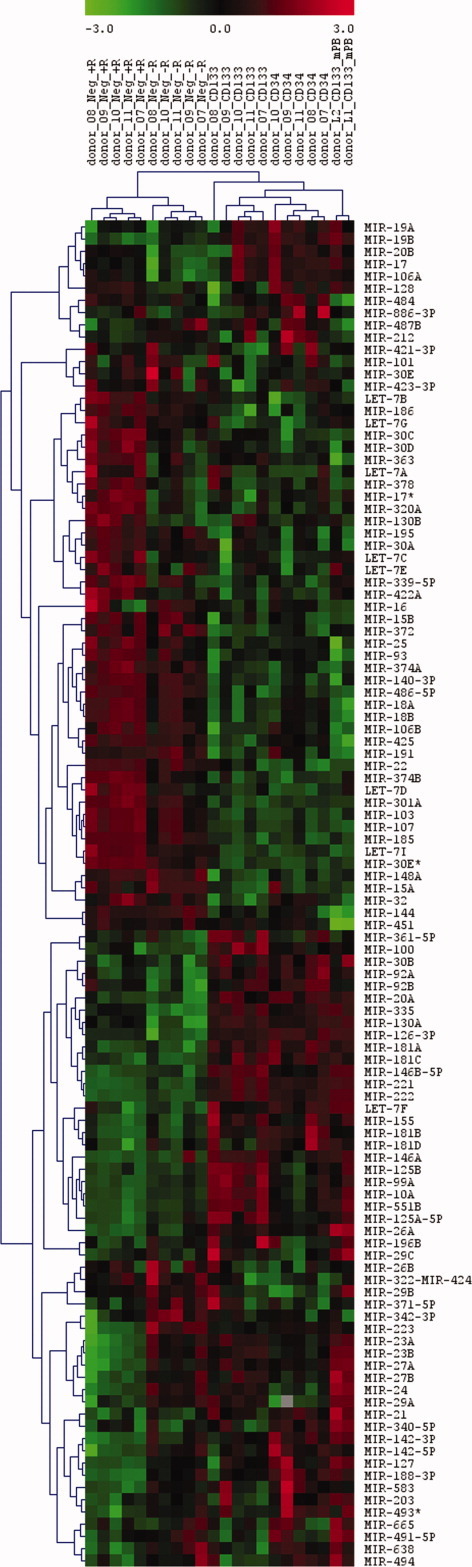
Average linkage cluster of miRNAs expressed in CD133^+^, CD34^+^CD133^−^, and CD34^−^CD133^−^ (Neg) cells. The cluster was obtained from array hybridizations of different bone marrow subpopulations (*n* = 5) and leukapheresis samples (mPB, *n* = 2) versus universal reference. Those miRNAs were included where the net signal intensity of the sample miRNA was onefold over background in at least four of five donors in the CD133^+^ and/or CD34^+^CD133^−^ cell population and where the miRNA was present in the UR. Neg +R: CD34^−^CD133^−^ with RBCs; Neg −R: CD34^−^CD133^−^ after RBC lysis. Log 2-transformed expression ratios are indicated from −3.0 (green) to 3.0 (red).

Next, we performed a cluster analysis of the miRNAs expressed in CD133^+^ and CD34^+^CD133^−^ cells. The four subpopulations clustered separately as visualized in Figure 2 and revealed distinct miRNA signatures for the different cell populations. As expected, the similarity between CD133^+^ and CD34^+^CD133^−^ cells was higher than between CD133^+^ and CD34^−^CD133^−^ cells which mainly consist of granulocytes (Neg −R) or RBCs (Neg +R).

The miRNA profiles of the CD133^+^ bone marrow samples and the CD133^+^ mobilized leukapheresis samples were highly similar with the notable exception of miR-451 and miR-144 expression that could only be detected in the samples derived from bone marrow and not in the leukapheresis samples.

**Figure 3:**
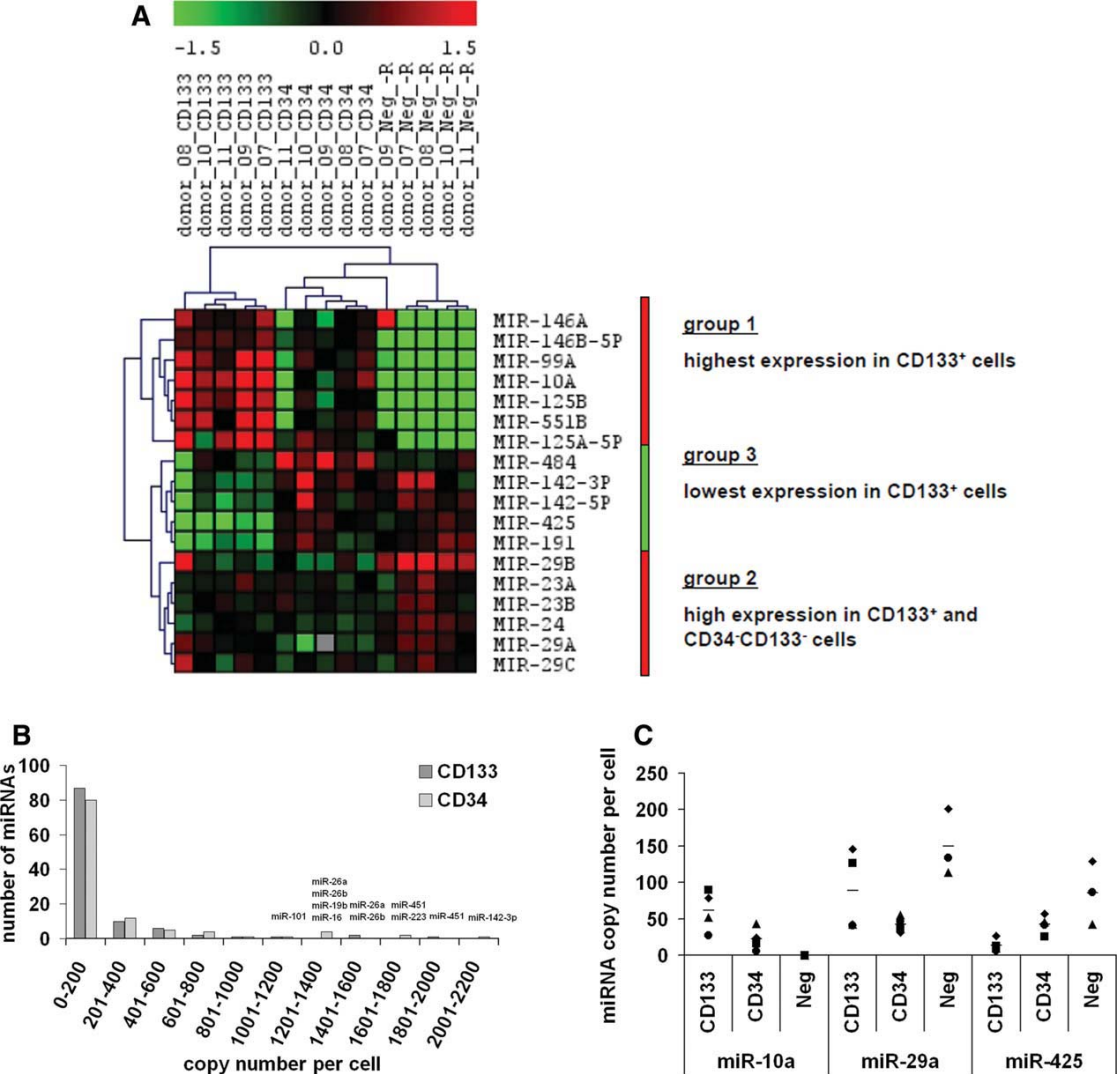
Relative and absolute expression of miRNAs in CD133^+^ cells. (**A**): Cluster analysis of miRNAs identified by discriminatory gene analysis of CD133^+^ cells compared with CD34^+^CD133^−^ cells. Discriminatory gene analysis was done by using the significance analysis of microarrays algorithm returning 18 miRNAs (false discovery rate (median): < 0.0001%) differentially expressed between CD133^+^ and CD34^+^CD133^−^ cells. The resulting miRNAs were grouped by similarities in expression patterns using two-dimensional hierarchical average linkage clustering. (**B**): Histogram displaying the number of miRNAs in distinct copy number intervals, for example, 87 of the miRNAs detected in CD133^+^ cells were present with less than 200 copy numbers per cell and 10 miRNAs with copy numbers between 201 and 400. (**C**): MicroRNA copy numbers prepared from four different donors (donor no. 7, 8, 10, and 11). The three miRNAs represent the three different expression signatures of the differentially expressed miRNAs: miR-10a (group1), miR-29a (group 2), and miR-425 (group 3). Neg: CD34^−^CD133^−^ cell subpopulation after red blood cell lysis (Neg −R).

SAM was performed to investigate the differences between CD133^+^ and CD34^+^CD133^−^ cells (Fig. 3A). Eighteen miRNAs were significantly differentially expressed (false discovery rate [median] < 0.0001%) with three distinct expression signatures: 13 miRNAs (group 1 and 2) were significantly higher expressed in CD133^+^ compared with CD34^+^CD133^−^ cells. These 13 miRNAs could be divided into one group of miRNAs that were not expressed in CD34^−^CD133^−^ cells (group1) and another group where the expression in CD34^−^CD133^−^ cells was even higher than in CD133^+^ cells (group 2). Five miRNAs (group 3) were significantly lower expressed in CD133^+^ cells as in CD34^+^CD133^−^ cells.

These three expression signatures were observed with both populations of CD34^−^CD133^−^ cells. The miRNAs in group 1 seemed to be most specific for HSCs and HPCs as they were not expressed in the bulk of bone marrow cells. The ratios for the 18 miRNAs significantly differentially expressed between CD133^+^ and CD34^+^CD133^−^ cells are shown in Supporting Information Table 2.

The microarray data was also used to calculate the miRNA copy numbers per cell for the hematopoietic cell populations of four different donors. This absolute quantification was possible as the samples were hybridized versus the UR and analyzed according to our previously published method for absolute quantification [26]. The miRNA copy numbers were calculated for all detected miRNAs being also present in the UR and Spearman correlation coefficients were determined to assess the overall performance and to show the relationship between the different samples. The Spearman correlation coefficients of the miRNA copy number expression profiles were 0.88–0.97 for the CD133^+^ cells and 0.90–0.95 for the CD34^+^CD133^−^ cells. A comparison of the CD133^+^ cells with the CD34^+^CD133^−^ cells led to correlation coefficients between 0.81 and 0.94 (Supporting Information Table 3). Taken together, the correlation coefficients revealed a high similarity between different donors and a robust absolute measurement of miRNAs.

Next, the miRNA copy numbers were analyzed in detail. The majority of all detected miRNAs, 79% in CD133^+^ cells and 73% in CD34^+^CD133^−^ cells, were present with less than 200 copies per cell, whereas the highest expressed miRNAs showed copy number of around 2,000 per cell (Fig. 3B). The most abundant miRNAs in CD133^+^ cells were miR-26a (7.2% of overall copy numbers), miR-451 (7.0%), and miR-26b (6.5%). The 25 highest expressed miRNAs in CD133^+^ and CD34^+^CD133^−^ cells accounted for 72.8% and 74.2%, respectively, of the overall miRNA pool (Supporting Information Table 4). However, these miRNAs are neither exclusively found in CD133^+^ and CD34^+^CD133^−^ cells nor are most of them differentially expressed between the two populations.

The significantly differentially expressed miRNAs (Fig. 3A) showed rather low expression levels with copy numbers below 200 copies per cell with exception of miR-142-3p with up to 5,000 copies per cell for one donor. Figure 3C displays the copy numbers of three exemplary miRNAs representing the three different expression signatures of the differentially expressed miRNAs in CD133^+^ cells. miR-10a was expressed at highest level in CD133^+^ cells (group 1), miR-29a at high level in CD133^+^ and CD34^−^CD133^−^ cells (group 2), and miR-425 at lowest level in CD133^+^ cells (group 3).

### Validation of miRNA Microarray Data

We validated our miRNA microarray results using hybridization-independent methods, small RNA cDNA library sequencing and quantitative (q) RT-PCR.

Small RNA cDNA libraries were generated from 2 μg of total RNA of CD133^+^ and CD34^+^CD133^−^ cells from donor 7 and 10 and Solexa sequenced. To allow for the quantification of the total miRNA levels, 5 fmol of a cocktail of synthetic calibrator sequences without a match to the human genome were spiked into the samples. For each of the small RNA, cDNA libraries between 210,000 and 300,000 sequence reads were obtained and of these about 67% were annotated as miRNAs. We focused on the robustly expressed ones and considered only miRNAs showing a minimum of 100 sequence reads.

**Figure 4:**
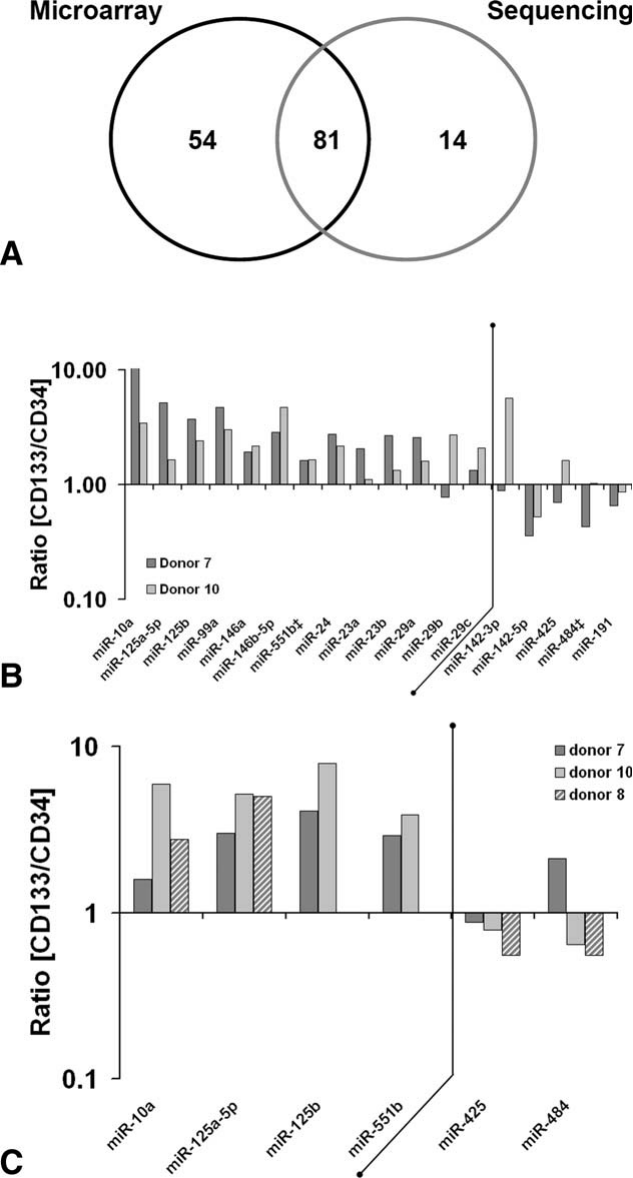
Validation of microarray data via Solexa sequencing and quantitative real-time polymerase chain reaction (qRT-PCR). (**A**): Venn diagram showing miRNAs detected with over 100 counts (sequencing) or onefold over background (array data) in CD133^+^ and/or CD34^+^CD133^−^ cells. (**B**): The ratios of CD133^+^ counts versus CD34^+^CD133^−^ counts for the 18 significantly differentially expressed miRNA are displayed for two donors. miRNAs that were present with less than 100 counts are marked with ‡. (**C**): The expression levels of six miRNAs were also determined by qRT-PCR using the miScript system for two (miR-125b, miR-551b) or three donors. RN5S1 was used for normalization.

Ninety-five miRNAs were sequenced with over 100 counts compared with 135 miRNAs detected onefold over background on all arrays. The overlap between the two groups was 81 miRNAs detected with both methods (Fig. 4A).

Comparison of sequencing and microarray hybridization for the miRNAs detected with both methods led to Spearman correlation coefficients of 0.74 (CD34^+^CD133^−^ cells of donor 7), 0.73 (CD34^+^CD133^−^ cells of donor 10), 0.82 (CD133^+^ cells of donor 7), 0.71 (CD133^+^ cells of donor 10), and at least 0.94 within each method.

The relative frequencies of miRNA sequence read numbers of the 18 miRNAs detected as significantly differentially expressed between CD133^+^ and CD34^+^CD133^−^ samples (Fig. 3A) very well reflect the microarray results (Fig. 4B). Twelve of thirteen higher expressed and four of five lower expressed miRNAs were validated for donor 7, for donor 10 all higher expressed miRNAs and two of the lower expressed miRNAs were validated. Sequencing data revealed six further miRNAs that were expressed higher in CD133^+^ cells as compared with CD34^+^CD133^−^ cells, namely, miR-99b, miR-144*, let-7c, miR-181d, miR-22, and miR-222. Additionally, miR-128 was found to be lower expressed in CD133^+^ cells.

As a further control, qRT-PCR was performed for six arbitrary selected miRNAs (Fig. 4C). The results correlated well with the microarray and the sequencing-based approaches.

### Combined miRNA and mRNA Analysis

To further analyze the role of the differentially expressed miRNAs in CD133^+^ stem cells, we generated mRNA microarray expression profiles (Supporting Information Table 5 and Supporting Information Fig. 2) and examined the coexpression of bioinformatically predicted miRNA-mRNA pairs. Two main approaches for the combined miRNA and mRNA analysis were used (Fig. 1B). The first one focused on potential target mRNAs that were expressed in the same cell population as the miRNAs of interest. Thereby, potential miRNA actions, where the effect of a miRNA on the mRNA level of its target is quite modest and therefore, not measurable are also considered. In contrast, the second approach focused on inversely expressed miRNAs and mRNAs.

For the first analysis, target prediction was carried out for each of the groups in Figure 3A separately considering targets that were predicted by at least three target prediction tools. Subsequently, the mRNA expression profiles were used to select the expressed mRNAs among the predicted mRNAs followed by an annotation enrichment analysis. For the seven miRNAs (miR-146b-5p, miR-125a-5p, miR-125b, miR-99a, miR-146a, miR-10a, miR-551b) higher expressed in CD133^+^ cells compared with CD34^+^CD133^−^ cells and CD34^−^CD133^−^ cells (Supporting Information Tables 6 and 7) annotation groups such as insulin signaling, B-cell receptor signaling, and positive regulation of cell differentiation were enriched. Most interestingly, the predicted targets for the miRNAs higher expressed in CD133^+^ cells were also enriched for the GO category “hematopoietic or lymphoid organ development” which comprises transcription factors such as ETS1, KLF11, and HOXB3.

The targets predicted for the second group of higher expressed miRNAs in CD133^+^ cells (miR-29a, miR-29b, miR-29c, miR-23a, miR-23b, miR-24) were enriched for annotations related to developmental processes, the plasma membrane-bound protein complex SNARE and negative regulation of cell migration (Supporting Information Tables 6 and 7).

The targets for the lower expressed miRNAs, that is, miR-142-3p, miR-142-5p, miR-484, miR-425, and miR-191, in CD133^+^ cells showed a high enrichment for the ubiquitin proteasome system (UPS) (Supporting Information Tables 6 and 7).

In conclusion, the annotation analysis indicated that the significantly differentially expressed miRNAs in CD133^+^ cells play a role in maintaining the stem cell character: The higher expressed miRNAs target mRNAs involved in cell differentiation and may prevent differentiation, whereas the lower expressed miRNAs target relevant mRNAs for the UPS that is known to be involved in the fine-tuning of HSC homeostasis [38].

In a second approach (Fig. 1B), we analyzed inversely expressed miRNAs and mRNAs and found 46 mRNAs that were lower expressed in CD133^+^ cells and targets of the 13 higher expressed miRNAs in CD133^+^ cells (Supporting Information Table 8). A functional grouping analysis for the 46 predicted and lower expressed mRNAs revealed a significant enrichment of biological processes related to development, cellular import/export, cell differentiation, cytoskeleton, and cell migration (Supporting Information Fig. 3). An interesting example for the 46 predicted and lower expressed targets was FZD5, a receptor of the Wnt-signaling pathway, and TPM1, an actin binding protein, both predicted targets of miR-29a.

For the lower expressed miRNAs and higher expressed mRNAs in CD133^+^ cells just eight miRNA-mRNA pairs were found (Supporting Information Table 9). Interestingly, the miRNA-mRNA pair miR-142-3p and CD133 was one of the predictions suggesting that a low miR-142-3p expression might allow higher expression of CD133 in CD133^+^ progenitor cells.

### In Vitro Characterization of Functional miRNA and mRNA Interactions

**Figure 5:**
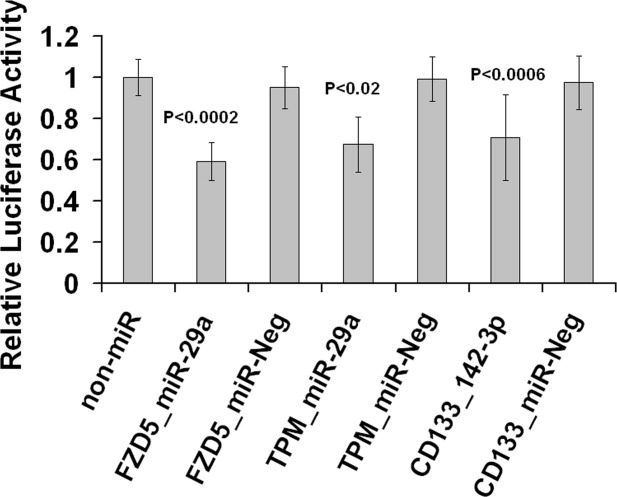
Verification of miRNA-mRNA interactions via luciferase assay. Cotransfection of pMIR-Luc-FZD5 and 100 nM miR-29a decreased 41% of the luciferase activity in comparison with the blank control. Cotransfection of pMIR-Luc-TPM and miR-29a decreased the luciferase activity by 33% and pMIR-Luc-CD133 and miR-142-3p by 30%. For each cotransfection, 300 ng vector and 50 nM (miR-142-3p) or 100 nM (miR-29a) oligonucleotide were used. Relative luciferase activity represents *firefly* luciferase activity normalized versus *renilla* activity. Data are representative of at least five independent experiments. miR-Neg, oligonucleotide designed to serve as negative control.

Luciferase reporter-based assays were used to analyze some of the miRNA-mRNA interactions. In detail, we cloned the respective miRNA-binding site region of the FZD5, TPM1, and CD133 3′UTRs behind a luciferase reporter gene (Fig. 5 and Supporting Information Fig. 4). Direct interactions of miRNAs and putative target sites are then assessed based on selective degradation or translational repression of luciferase target gene 3′UTR fusion transcripts.

Cotransfection of 100 nM miR-29a and 300 ng pMIR-Luc-FZD5 decreased the luciferase activity by 40% and an even higher decrease of 60% was achieved by decreasing the vector amounts (Supporting Information Fig. 4). Besides miR-29a, binding sites for miR-24 and miR-99a were predicted within the FZD5 3′UTR. We also tested their potential function, but a reproducible decrease in luciferase activity was not detectable for either of the tested miRNAs (data not shown).

The activity of pMIR-Luc-TPM1 was decreased by 35% upon cotransfection with miR-29a and again an even higher decrease of 58% was observed using lower vector amounts. The negative control using pMIR-Luc without cloned target sequence showed no decrease in luciferase activity after cotransfection with miR-29a.

The influence of miR-142-3p, that was described as hematopoietic specific [27], on pMIR-Luc-CD133 was less pronounced, the luciferase activity was decreased by 20%–30%. Interestingly, this effect was achieved reproducibly just with 50 nM miR-142-3p mimic and not with 100 nM miR-142-3p.

In conclusion, the luciferase assays indicated that FZD5, a receptor of the Wnt-signaling pathway, and TPM1, which are lower expressed in CD133^+^ cells, are controlled by miR-29a. The expression of CD133 is probably regulated by the hematopoietic-specific miRNA miR-142-3p.

### Influence of miR-142-3p and miR-425 on Proliferation and Differentiation of CD133^+^ Cells

The influence of miR-142-3p and miR-425 on CD133^+^ cells was analyzed by performing the CFSE staining method and CFU assays.

**Figure 6:**
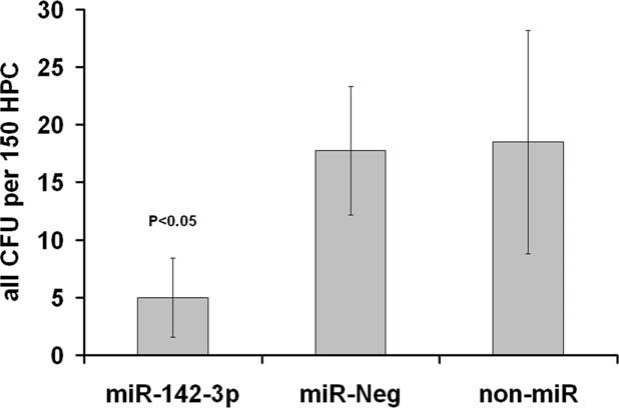
miR-142-3p inhibits colony formation by CD133^+^ cells. CD133^+^ cells were transfected with 50 nM miR-142-3p or control (miR-Neg) and cultivated for 7 days. After 7 days of cultivation, the progeny (150 CD34^+^ cells per 1 ml) was seeded in methylcellulose medium. The overall colony-forming ability was decreased by 71% (*n* = 4). Abbreviations: CFU, colony-forming unit; HPC, hematopoietic progenitor cell.

CD133^+^ cells were stained with CFSE and transfected with miR-142-3p, miR-425, or a control miRNA (miR-Neg) and cultivated for 6 days. The fluorescence dye CFSE is precisely halved at each cell generation, and therefore, proliferation is associated with reduced CFSE signal intensity. miR-142-3p as well as miR-425 led to a slightly but significantly decreased proliferation of CD133^+^ cells (*p* < .01 and *p* < .02, respectively, see Supporting Information Fig. 5). Notably, CD133^+^ cells transfected with miR-142-3p showed a decrease in the overall colony-forming ability of 71% (Fig. 6), whereas miR-425 decreased the percentage of CFU-M from 20% to 8.5% (Supporting Information Fig. 6).

## DISCUSSION

The miRNA signature comprising relative and absolute expression levels of CD133^+^ cells was determined by microarrays, qRT-PCR, and Solexa sequencing. On this basis, we selected differentially expressed miRNAs in CD133^+^ cells in comparison with CD34^+^CD133^−^ cells.

CD133 appear to be ancestral to CD34, as CD34^+^ cells can be generated in vitro from CD133^+^CD34^−^cells [15]. To elucidate the molecular architecture of CD133^+^ cells and the role of miRNAs in the early steps of hematopoiesis, we compared CD133^+^ cells with CD34^+^CD133^−^ cells. It is noteworthy that most of the CD133^+^ cells (>99%) express CD34 and half of the CD34^+^ cells express CD133 [13].

Currently, the available miRNA profiles for human HSCs and HPCs are restricted to CD34^+^ cells from bone marrow, peripheral blood, mobilized peripheral blood and cord blood [9, 10, 27], CD34^+^CD38^−^ cells from cord blood [5, 6], and CD133^+^ cells from mobilized peripheral blood [39]. To our knowledge, here, we compared for the first time CD133^+^ and CD34^+^CD133^−^ bone marrow cells from the same donor on miRNA and mRNA level. Most of the miRNAs (79%) in CD133^+^ cells were present with less than 200 copies per cell and the 25 highest expressed miRNAs accounted for 72.8% of the miRNA pool. A finding that correlates well with previously published sequencing data [40]. The majority of miRNAs was expressed at low levels, whereas only a few miRNAs were expressed at high level with around 2,000 copies per cell (Fig. 3B), for example, miR-26a amounted to 7.2% of the overall miRNA pool. Although miR-26a was expressed at high levels, it is possible that the majority of low-expressed miRNAs are of biological significance when acting in combination with other low-expressed miRNAs [41].

A combined miRNA and mRNA analysis was performed to develop a model for the biological role of the 18 differentially expressed miRNAs in CD133^+^ stem cells (Fig. 3A). The mRNA data used for filtering of the predicted targets was generated from the same cells as the miRNA profiles.

**Figure 7:**
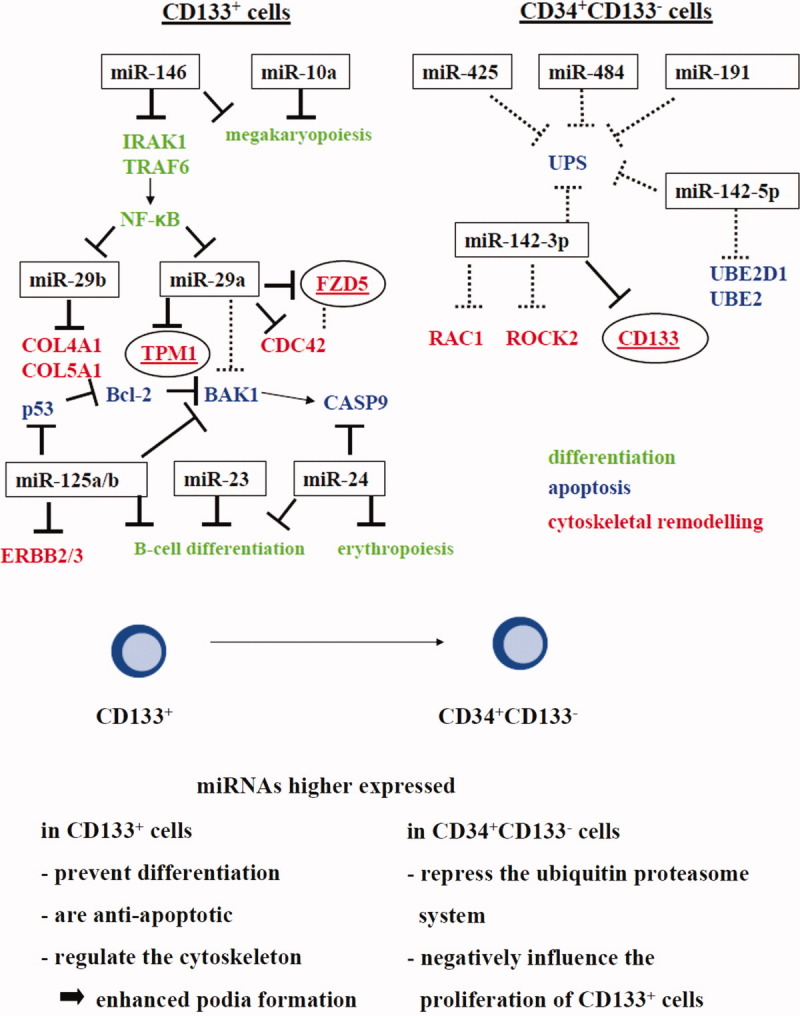
Model for the biological role of significantly differentially expressed miRNAs in CD133^+^ cells. (**A**): The targets of the miRNAs are highly relevant in the context of differentiation (green), apoptosis (blue), and cytoskeletal remodeling (red). Predicted miRNA-mRNA interactions are shown as dashed line. Targets that were validated in our laboratory are encircled (Fig. 5). The references for the other validated miRNA-mRNA interactions are mentioned in the text. (**B**): Hypothesis for the biological role of miRNAs in CD133^+^ cells. Abbreviations: BAK1, Bcl-2 antagonist killer 1; CASP9, caspase 9; FZD5, frizzled homolog 5; IRAK1, interleukin-1 receptor–associated kinase 1; NF-κB, nuclear factor of κ light polypeptide gene enhancer in B-cells 1; TRAF 6, TNF receptor–associated factor 6; UPS, ubiquitin proteasome system.

Notably, mRNAs expressed in CD133^+^ cells and predicted to be targeted by miRNAs higher expressed in CD133^+^ cells as compared with CD34^+^CD133^−^ and CD34^−^CD133^−^ cells (group 1; Fig. 3A) were enriched for the GO category “hematopoietic or lymphoid organ development.” Interestingly, the corresponding miRNAs have already been described to play a role in hematopoiesis. It was shown that miR-10a [42], miR-146a [43], and miR-125b [44] inhibit differentiation of specific hematopoietic lineages (Fig. 7A).

Furthermore, p53 and the proapoptotic B-cell CLL/lymphoma 2 antagonist killer 1 (BAK1) are targeted by miR-125b, suppressing apoptosis in human cancer cells [45, 46]. BAK1 is also directly downregulated by miR-125a [47]. In addition, suppression of v-erb-b2 erythroblastic leukemia viral oncogene homolog 2 (ERBB2) and ERBB3 by enforced expression of miR-125a or miR-125b resulted in impaired anchorage-dependent growth in breast cancer cells [48]. As activation of ERBB2 can induce loss of cell polarity, suppression of ERBB2 by miR-125 in CD133^+^ cells might lead to a more polarized morphology.

Taken together, miR-146, miR-10a, and miR-125b might prevent differentiation, whereas miR-125b seems to play an antiapoptotic role in CD133^+^ cells as well as taking part in remodeling of the cytoskeleton (Fig. 7A).

The second group of higher expressed miRNAs in CD133^+^ cells compared with CD34^+^CD133^−^ cells were also highly expressed in CD34^−^CD133^−^ cells (group 2; Fig. 3A) and were among others enriched for annotations related to developmental processes, negative regulation of cell migration and collagens. Roles for the respective miRNAs in developmental processes were already shown for miR-23a, miR-23b, miR-29a, and miR-24 [49–51]. Furthermore, an influence of miR-29b on the regulation of the extracellular matrix and of miR-24a on the proapoptotic factor caspase 9 was demonstrated [52, 53].

The predicted targets of the lower expressed miRNAs (group 3; Fig. 3A) were highly enriched for the UPS and the regulation of cytoskeleton organization. Interestingly, a function of miR-142-5p in ubiquitin-mediated proteolysis and of miR-142-3p in regulation of cytoskeleton was also predicted by Tsang et al. [54] using the computational method mirBridge.

After analyzing the coexpression of bioinformatically predicted miRNA-mRNA pairs (approach I; Fig. 1B), we focused on inversely correlated miRNA-mRNAs pairs (approach II; Fig. 1B) and analyzed some of them via luciferase assays. One of those pairs was miR-29a and TPM1, an important component of the cytoskeleton playing a fundamental role in many aspects of eukaryotic cell behavior such as cell morphology, divisions, and motility. Our results revealed that TPM1 can be regulated by miR-29a and that this interaction could lead to a lower expression of TPM1 in CD133^+^ cells. As tropomyosin inhibits lamellipodium formation [55], a lower expression of TPM1 in CD133^+^ cells might lead to enhanced podia formation as compared with CD34^+^CD133^−^ cells. Interestingly, it was described that more primitive HPCs possess more lamellipodia with strong expression of CD133 [56].

Another inversely correlated miRNA-mRNA interaction was miR-29a and FZD5, one of the seven-pass transmembrane Frizzled receptors, which bind Wnt proteins. Wnt signaling controls diverse processes such as cell proliferation and stem cell maintenance. A role for Wnt signaling in self-renewal of HSCs was demonstrated by Reya et al. [57]. Regulation of cell polarity and migration by ß-catenin-independent Wnt pathways was also described [58].

Another very interesting interaction that was validated via luciferase assay was the binding of miR-142-3p, being lower expressed in CD133^+^ cells than in CD34^+^CD133^−^ cells, to the CD133 mRNA. Our results also provided first evidence that miR-142-3p has a negative influence on the proliferation of CD133^+^ cells. However, further analysis is necessary to show if the surface expression of CD133 is controlled by miRNAs.

According to the biological effects of the miRNAs summarized above (Fig. 7A), the miRNAs higher expressed in CD133^+^ cells prevent differentiation, are antiapoptotic and regulate the cytoskeleton leading to enhanced podia formation and cell polarity (Fig. 7B). The miRNAs higher expressed in CD34^+^CD133^−^ cells repress the ubiquitin-proteasome system and negatively influence the proliferation of CD133^+^ cells. Taken together, it is proposed that the miRNAs significantly differentially expressed in CD133^+^ cells as compared with CD34^+^CD133^−^ function by fine-tuning the expression of their targets to a precise level at which the gene will execute its specific function to prevent processes such as differentiation and apoptosis. In addition, they play a role in remodeling of the cytoskeleton. Detailed functional analysis of miRNA effects in CD133^+^ cells will gain further insights into the role of miRNAs in stem cells.

## CONCLUSION

We have presented the first relative and absolute miRNA profile of CD133^+^ bone marrow cells and the first comparison of donor-matched CD133^+^ and CD34^+^CD133^−^ cells on miRNA level. Our results have provided evidence that the miRNAs differentially expressed between the CD133^+^ and CD34^+^CD133^−^ cells are involved in inhibition of differentiation, proliferation, prevention of apoptosis, and cytoskeletal remodeling.

These findings extend our knowledge about the molecular architecture of CD133^+^ cells that are used in regenerative medicine as stem cell-based therapy for tissue regeneration.
