# Supplementary material for: Combined Characterization of microRNA and mRNA Profiles Delineates Early Differentiation Pathways of CD133+ and CD34+ Hematopoietic Stem and Progenitor Cells
Source: Stem Cells. 2011 Mar 10;29(5):847–57. doi: 10.1002/stem.627 (PMC3116150; doi:10.1002/stem.627)
Supplement: Supplementary file 1 [file stem0029-0847-SD1.doc]

**Supplemental data**

**GEO:**

The microarray data are deposited in the public repository Gene Expression Omnibus: http://www.ncbi.nlm.nih.gov/geo/query/acc.cgi?token=vtunpkoayuwmets&acc=GSE22460

**Table S1**

**Table S1. CliniMACs separation of CD133+ and CD34+CD133- cells from bone marrow.** The RNA was isolated according to the miRNeasy protocol. BM = bone marrow; f = female; m = male; oriori = percentage of CD133^+^ cells in original fraction before sample processing.

**Figure S1**

**Figure S1. Number of miRNAs expressed in the different hematopoietic subpopulations**. Venn diagram displaying distinct and shared miRNA expression in CD133^+^, CD34^+^CD133^–^ and CD133^–^CD34^–^ cells. For the CD34^–^CD133^–^ cells two sample types are shown, without (Neg +R) or with (Neg –R) RBC lysis. The miRNAs that were expressed once as high as the background intensity in at least four out of five experiments are taken into account.

**Table S2**

**Table S2. miRNAs significantly differentially expressed in CD133^+^ cells compared to CD34^+^CD133^–^.** The ratios were calculated by dividing the ratio of sample signal and UR signal of the CD133^+^ arrays and CD34^+^CD133^–^ arrays and display the x-fold stronger or weaker expression of a miRNA in the CD133^+^ cells as compared to the CD34^+^CD133^–^ cells. miRBase accession = MIMAT accession number.

| **miRNA**  **name** | **miRBase accession** | **Donor 7** | **Donor 8** | **Donor 9** | **Donor 10** | **Donor 11** | **mean** |
| --- | --- | --- | --- | --- | --- | --- | --- |
| miR-10a | 0000253 | 3.2 | 5.1 | 8.8 | 3.2 | 8.0 | 6.3 |
| miR-125b | 0000423 | 3.2 | 4.8 | 6.4 | 2.4 | 5.3 | 4.7 |
| miR-146a | 0000449 | 2.6 | 3.2 | 3.5 | 2.1 | 6.7 | 3.9 |
| miR-125a-5p | 0000443 | 4.9 | 6.0 | 3.6 | 0.6 | 2.8 | 3.2 |
| miR-551b | 0003233 | 3.2 | 3.5 | 4.4 | 3.7 | 3.2 | 3.7 |
| miR-99a | 0000097 | 2.9 | 5.0 | 4.2 | 2.1 | 2.6 | 3.5 |
| miR-29a | 0000086 | 2.0 | 3.2 | - | 4.7 | 1.5 | 3.1 |
| miR-146b-5p | 0002809 | 2.1 | 2.2 | 1.9 | 2.1 | 2.2 | 2.1 |
| miR-29b | 0000100 | 1.5 | 3.4 | 2.0 | 2.4 | 0.7 | 2.1 |
| miR-29c | 0000681 | 1.5 | 3.0 | 2.2 | 1.7 | 1.0 | 2.0 |
| miR-23a | 0000078 | 1.3 | 1.9 | 1.9 | 1.8 | 0.9 | 1.6 |
| miR-24 | 0000080 | 1.4 | 1.5 | 1.6 | 1.8 | 1.1 | 1.5 |
| miR-23b | 0000418 | 1.7 | 1.5 | 1.2 | 1.8 | 1.0 | 1.4 |
| miR-142-5p | 0000433 | 0.9 | 0.7 | 0.9 | 0.5 | 0.5 | 0.7 |
| miR-191 | 0000440 | 0.8 | 0.8 | 0.7 | 0.5 | 0.6 | 0.6 |
| miR-142-3p | 0000434 | 1.0 | 0.4 | 0.8 | 0.5 | 0.6 | 0.6 |
| miR-484 | 0002174 | 0.5 | 0.5 | 0.4 | 1.2 | 0.4 | 0.6 |
| miR-425 | 0003393 | 0.6 | 0.5 | 0.5 | 0.5 | 0.3 | 0.4 |

**Table S3**

**Table S3. Spearman correlation coefficients for the miRNA copy number expression profiles.** The miRNA copy numbers were calculated for all detected miRNAs (listed in Figure 2) and the coefficients were determined between the different samples.

|  |  | **CD133^+^ cells** | | | | **CD34^+^CD133^–^ cells** | | | |
| --- | --- | --- | --- | --- | --- | --- | --- | --- | --- |
|  |  | **Donor 7** | **Donor 8** | **Donor 10** | **Donor 11** | **Donor 7** | **Donor 8** | **Donor 10** | **Donor 11** |
| **CD133^+^**  **cells** | **Donor 7** | - | 0.93 | 0.97 | 0.94 | 0.94 | 0.92 | 0.91 | 0.84 |
|  | **Donor 8** | 0.93 | - | 0.92 | 0.88 | 0.90 | 0.90 | 0.85 | 0.81 |
|  | **Donor 10** | 0.97 | 0.92 | - | 0.96 | 0.92 | 0.93 | 0.94 | 0.89 |
|  | **Donor 11** | 0.94 | 0.88 | 0.96 | - | 0.90 | 0.90 | 0.93 | 0.93 |
| **CD34^+^CD133^–^ cells** | **Donor 7** | 0.94 | 0.90 | 0.92 | 0.90 | - | 0.95 | 0.92 | 0.90 |
|  | **Donor 8** | 0.92 | 0.90 | 0.93 | 0.90 | 0.95 | - | 0.95 | 0.92 |
|  | **Donor 10** | 0.91 | 0.85 | 0.94 | 0.93 | 0.92 | 0.95 | - | 0.92 |
|  | **Donor 11** | 0.84 | 0.81 | 0.89 | 0.93 | 0.90 | 0.92 | 0.92 | - |

**Table S4**

**Table S4. 25 most abundant miRNAs detected in CD133^+^ and/or CD34^+^CD133^–^ cells.** The percentages of the overall copy numbers for the most abundant miRNAs are shown, calculated as the median value of four independent measurements. These most abundant miRNAs are not exclusively found in CD133^+^  and CD34^+^CD133^–^ cells.

| **CD133 miRNAs [%]** | | **CD34 miRNAs [%]** | |
| --- | --- | --- | --- |
| miR-26a | 7.2 | miR-142-3p | 8.5 |
| miR-451 | 7.0 | miR-451 | 5.9 |
| miR-26b | 6.5 | miR-223 | 5.4 |
| miR-142-3p | 5.7 | miR-26b | 5.1 |
| miR-16 | 4.7 | miR-16 | 4.8 |
| miR-101 | 4.6 | miR-101 | 4.6 |
| miR-19b | 4.2 | miR-26a | 4.5 |
| miR-223 | 4.0 | miR-19b | 4.5 |
| let-7f | 2.8 | miR-20a | 2.8 |
| miR-20a | 2.4 | miR-142-5p | 2.8 |
| miR-222 | 2.1 | let-7f | 2.6 |
| miR-92a | 2.0 | miR-92a | 2.1 |
| miR-19a | 2.0 | miR-19a | 2.1 |
| miR-142-5p | 2.0 | miR-15a | 2.0 |
| miR-15a | 1.9 | miR-30b | 2.0 |
| miR-30b | 1.9 | miR-21 | 2.0 |
| miR-21 | 1.9 | miR-144 | 1.9 |
| miR-126-3p | 1.5 | miR-126-3p | 1.8 |
| miR-92b | 1.4 | miR-92b | 1.5 |
| let-7a | 1.3 | miR-222 | 1.4 |
| miR-144 | 1.3 | miR-17 | 1.4 |
| miR-17 | 1.2 | miR-181a | 1.1 |
| miR-29c | 1.1 | let-7a | 1.0 |
| miR-221 | 1.1 | miR-20b | 1.0 |
| miR-20b | 1.0 | let-7g | 1.0 |

**Table S5 provided as Excel file**

**Table S5. Significantly differentially expressed transcripts between CD133^+^ and CD34^+^CD133^–^ cells.** The transcripts were identified by using SAM analysis (false discovery rate: 0.000000%) and t-test (p-value < 0.01). See Excel file.

**Figure S2**

**Figure S2. Average linkage cluster of mRNAs that are significantly differentially expressed between CD133^+^ and CD34^+^CD133^–^ cells.** Discriminatory gene analysis by the SAM algorithm resulted in 80 mRNAs that are differentially expressed. Neg_L = Neg –R.

**Table S6**

**Table S6. Combined miRNA and mRNA analysis using approach I** (see Table 1 in main part). 1) miRNA groups (as described in Figure 3A) used as input for target prediction tools. 2) Targets that were predicted by at least three prediction tools were considered. 3) Only those predicted targets were considered that were detected on Agilent Whole Human Genome Oligo Microarrays. 4) The predicted and detected targets for the respective miRNAs were used as input for AEA using TreeRanker software. Details for the enriched categories are listed in Table S7.

**Table S7**

**Table S7. Significantly enriched categories among the predicted mRNA targets for the significantly differentially expressed miRNAs.**

(A) Significantly enriched categories among the predicted mRNA targets for the significantly differentially expressed miRNAs miR-146a, miR-146b-5p, miR-125a-5p, miR-125b, miR-99a, miR-10a, and miR-551b (strategy Ia, group 1 in Table S6). Gene bank numbers are listed in Table S10.

| **Category and the respective genes** | **Count** | **Significance** | **EF** |
| --- | --- | --- | --- |
| **insulin Signaling** (Wiki pathways, GenMAPP pathways) | 11 | 2.90E-06 | 5.94 |
| GRB10; FRAP1; IGF1R; MAPK14; MAP3K7; FLOT2; MAP3K11; MAP3K10; MAP2K7; RPS6KA1; RHOQ |  |  |  |
| **BCR signaling** (PathwayCommons, Nature pathways) | 8 | 5.00E-06 | 8.42 |
| ETS1; MAPK14; CAMK2G; MAP3K7; CSNK2A1; TRAF6; PPP3CA; CSNK2A1 |  |  |  |
| **hematopoietic or lymphoid organ development** (GO) | 17 | 1.80E-05 | 3.51 |
| HOXB3; HOXA3; PURB; BAK1; ESRRA; B3GAT2; BCL2; JARID2; HMGB3; NCOA6; BCL2L11; ETS1; ZFP36; MED1; MAPK14; KLF11; TRAF6 ; |  |  |  |
| **nuclear chromosome part** (GO) | 11 | 4.00E-05 | 5.22 |
| PURB; UBE2I; H3F3B; BAZ2A; PHC1; XRN1; ORC2L; SUV39H1; SUV420H2; CSNK2A1; CSNK2A1 |  |  |  |
| **PHD** (homology domain) | 10 | 8.50E-05 | 7.4 |
| BAZ2B;A_23_P120644; DPF2; PHF20L1; PHF15; BAZ2A; BRPF1; ZMYND11; FBXL10; MLL2 |  |  |  |
| **MAPKKK cascade** (GO) | 14 | 9.30E-05 | 4.22 |
| SEMA4C; FGFR3; CDC42SE1; IGF1R; MAPK14; MAP3K7; IRAK1; TRAF6; RB1CC1; MAP3K11; SHANK3; MAPKBP1; MAP3K10; MAP2K7 ; |  |  |  |
| **positive regulation of cell differentiation** (GO) | 9 | 9.57E-03 | 3.41 |
| B3GAT2; TIAM1; BCL2; FGFR3; FNDC3B; ETS1; MAPK14; NUMB; SEMA4D |  |  |  |

(B) Significantly enriched categories among the predicted mRNA targets for the significantly differentially expressed miRNAs miR-29, miR-23 and miR-24 (strategy Ia, group 2 in Table S6). Gene bank numbers are listed in Table S10.

| **Category and respective genes** | **Count** | **Significance** | **EF** |
| --- | --- | --- | --- |
| **reproductive developmental process (GO)** | 23 | 5E-09 | 4.49 |
| STAT5B; CCND2; FEM1B; HMGB2; CDKN1B; CSDA; HMGCR; BCL2L11; FOXO3; PLAG1;BCL2L2; NCOA4; PTEN; ROBO2; VEGFA; NKX2-1; CCND1; HNF1B; CHD7; CSDA; LGR4; MMP2; IDH1 |  |  |  |
| **sensory organ development (GO)** | 26 | 6.89E-08 | 3.83 |
| SPRY2; NUDT6; COL5A1; MAFB; ZEB1; ZFP91; CDKN1B; INSIG1; TSHZ1; BCL2L11; MAF; BHLHE22; MITF; PVRL1; SPARC; PRKRA; MUTED; MAB21L2; VEGFA; YY1; MEIS1; ZFP91; CHD7; KCNMA1; KLF4; TSPAN12 |  |  |  |
| **SNARE complex, SNAP receptor activity, SNARE interactions in vesicular transport (GO**) | 11 | 4.10E-06 | 10.38 |
| VTI1A; VTI1B; SCAMP5; STX1A; STX17; SNAP25; STX5; STX12; BET1; BET1; STX16; |  |  |  |
| **negative regulation of cell migration (GO**) | 12 | 5.00E-06 | 4.52 |
| PNRC2; TRIB1; NUDT6; CDKN1B; TPM1; DLC1; PTEN; PDGFB; VCL; LOC728003; TPM1; ABHD2 |  |  |  |
| **ECM and collagens (cancermodules)** | 20 | 7.50E-06 | 3.06 |
| PMP22; COL6A3; ZEB1; COL4A2; TPM1; MARCKS; MLLT11; PLAU;  ADNP; BMP1; MAF; MRC2; COL4A1; FBN1; SPARC; PLAGL1; WDTC1; CXCL12; TPM1; MMP2 |  |  |  |
| **Serine/Threonine protein kinases, catalytic domain (homology domains, SMART)** | 27 | 1.00E-05 | 3.11 |
| MAP4K4; GRK5; MAP3K5; MARK3; NLK; AKT3; CDK6; SGK1; DYRK2; MAP3K3; MAP2K6; DYRK1A; NEK6; ICK; PIM1; CAMK2G; CLK3; PAK6; STK4; ADRBK1; CSNK2A2; PRPF4B; PAK2; LOC646214; CAMK1D; PRKG1; MAP2K4 |  |  |  |
| **protein kinase inhibitor activity (GO)** | 10 | 1.10E-05 | 5.48 |
| YWHAG; TRIB1; CDKN1B; GMFB; WNK1; PKIA; YWHAE; TRIB2; HEXIM1; LOC440917 |  |  |  |

(C). Significantly enriched categories among the predicted mRNA targets for the significantly differentially expressed miRNAs miR-484, miR-425, miR-191 and miR-142 (strategy Ib, group 3 in Table S6). Gene bank numbers are listed Table S10.

| **Category and respective genes** | **Count** | **Significance** | **EF** |
| --- | --- | --- | --- |
| **Ubiquitine proteasome system (Miltenyi)** | 39 | 5.13E-09 | 3.13 |
| ZBTB11; MARCH6; ASB7; PAFAH1B1; ZFAND3; SACS; MARK3; KIAA0368; RNF139; UBE2A; MYCBP2; CUL4A; RNF145; MAP3K7IP2; SENP1; BACH1; OTUD4; PCGF3; UBQLN1; TFG; ATXN7L2; CBL; FAM63B; ZBTB47; USP38; HECTD1; HGS; GNB2; WDR26; EPN2; RNF138; SH3RF1; UBE2D1; TRIM2; BTBD7; HLTF; SPOPL; SOCS6; RC3H1 |  |  |  |
| **regulation of cytoskeleton organization (GO)** | 10 | 4.30E-04 | 4.86 |
| MYCBP2; ROCK2; RAC1; TNKS; XPO1; CIT; ARHGEF2; MLL; RICTOR; RHOQ |  |  |  |
| **chromatin modification (GO)** | 14 | 2.69E-04 | 3.72 |
| MORF4L2; UBE2A; CHD9; MYST2; UTX; SATB1; PHF21A; SETD2; MLL; BAZ1A; HLTF; RCOR1; RERE; CBX6 |  |  |  |
| **protein serine/threonine kinase activity (GO)** | 15 | 6.20E-05 | 3.87 |
| HIPK1; MARK3; MYLK; CDK6; ROCK2; DYRK1A; PKN2; ZAK; OXSR1; CIT; STK38L; DYRK4; MAP3K11; SMG1; PRPF4B |  |  |  |
| **Reelin signaling pathway (PathwayCommons, Nature pathways)** | 4 | 8.60E-05 | 17.35 |
| PAFAH1B1; CBL; ARHGEF2; MAP3K11 |  |  |  |

**Figure S3**

**Figure S3. Biological processes term distribution frequency for mRNAs lower expressed in CD133^+^ cells and predicted to be a target of higher expressed miRNAs** (approach II, Table 1). The annotations used were derived from GO which provides information on molecular function. Highly relevant categories with p-values < 0.02 are marked with an asterisk.

**Table S8**

**Table S8. Lower expressed mRNAs in CD133^+^ cells predicted (by TargetScan) as a target of the higher expressed miRNAs.** The functional groups of the mRNAs are listed as well. Some of the miRNAs encompass a miRNA family as the predicted targets are often identical. miR-125: miR125a-5p and miR-125b; miR-29: miR-29a, miR-29b and miR-29c; miR-23: miR-23a and miR-23b.

**Table S9**

**Table S9. Higher expressed mRNAs in CD133^+^ cells predicted (by TargetScan) to be target of the lower expressed miRNAs.**

| **Target Gene Symbol** | **Target Gene Description** | **miR-142-3p** | **miR-425** |
| --- | --- | --- | --- |
| INPP5F | inositol polyphosphate-5-phosphatase F | X |  |
| COL24A1 | collagen, type XXIV, alpha 1 | X |  |
| MYO5A | myosin VA (heavy chain 12, myoxin) | X |  |
| NUDT11 | nudix (nucleoside diphosphate linked moiety X)-type motif 11 | X |  |
| ARID5B | AT rich interactive domain 5B (MRF1-like) | X |  |
| PROM1 (CD133) | prominin 1 | X |  |
| SH3RF1 | SH3 domain containing ring finger 1 |  | X |
| LOC283824 | hypothetical protein LOC283824 |  | X |

**Table S10 provided as Excel file**

**Table S10. List of Gene bank numbers.** Gene bank numbers (Entrez IDs) are listed in alphabetical order for the genes analyzed in the framework of the combined miRNAs and mRNA analysis. See Excel file.

**Figure S4**

**Figure S4. Verification of miRNA-mRNA interactions using different mimic (oligonucleotides) and vector amount**s. (A) Cotransfection of pMIR-Luc-FZD5 and miR-29a. (B) Cotransfection of pMIR-Luc-TPM and miR-29a. (C) Cotransfection of pMIR-Luc-CD133 and miR-142-3p Relative luciferase activity represents firefly luciferase activity normalized versus renilla acitivity. Data are representative of at least 3 independent experiments. Data with an asterisk (*) were performed in duplicates.

**Figure S5**

Figure S5. **Influence of miR-142-3p and miR-425 on proliferation and differentiation of CD133^+^ cells transfected with miRNAs.** CD133^+^ cells were stained with CFSE, transfected with miRNAs and cultured for 6 days. (A) The number of cell divisions and the percentage of cells in each division is indicated. CD133^+^ cells transfected with miR-142-3p contained a lower proportion of cells that passed through more than two cell divisions compared to cells transfected with miR-Neg. One representative histogram is shown. (B) Mean CFSE intensity of CD133^+^ cells was higher for cells transfected with miR-142-3p and miR-425 as compared to the negative controls. Presented significance values were determined in relation to miR-Neg (n=4). (C) Proportion of CD133^+^ cells in each cell division (n=4).

**Figure S6**

**Figure S6. Relative proportion of CFU-M.** CD133^+^ cells were transfected with miR-425 or control (miR-Neg) and seeded in methylcellulose medium. The percentage of CFU-M (colony forming unit – macrophage) decreased from 20% to 8.5%.
